# Commentary: The impact of palliative transurethral resection of the prostate on the prognosis of patients with bladder outlet obstruction and metastatic prostate cancer: A population-matched study

**DOI:** 10.3389/fsurg.2023.1123602

**Published:** 2023-01-23

**Authors:** Chao Lv, Qing Yuan, JingMin Yan, Bin Sun, Xu Zhang

**Affiliations:** ^1^ Department of Urology, PLA Air Force Medical Center, Beijing, China; ^2^ Department of Urology, 3rd Medical Center of PLA General Hospital, Beijing, China

**Keywords:** palliative transurethral resection of the prostate, metastatic prostate cancer (mPCa), bladder outlet obstruction (BOO), overall surivival 总生存率, cancer specific survival (CSS)

A Commentary on The impact of palliative transurethral resection of the prostate on the prognosis of patients with bladder outlet obstruction and metastatic prostate cancer: A population-matched study By Lv C, Yuan Q, Yan JM, Sun B and Zhang X. (2023) Front Surg. 10: 1123602. doi:10.3389/fsurg.2023.1123602

## Introduction

We are pleased to go through the manuscript by Fang et al., published in Frontiers in Surgery, which focuses on the prognosis of palliative transurethral resection of the prostate (pTURP) in the treatment of metastatic prostate cancer (mPCa) with bladder outlet obstruction (BOO), as he brings up an excellent discussion topic (1). The data were obtained from the Surveillance, Epidemiology, and End Results (SEER) database. A propensity score match (PSM) (1:1) was used to balance covariates, such as demographic characteristics, oncology features and cancer treatment. There were 1942 patients each in the pTURP and no-surgery groups. The results showed that the OS (overall survival) and CSS (cancer-specific survival) in the pTURP group were significantly lower than the non-surgical group (36.49 ± 0.94 vs. 45.52 ± 1.23 months in OS and 50.1 ± 1.49 vs. 61.28 ± 1.74 months in CSS). Overall mortality (HR: 1.19, *p* < 0.001) and cancer-specific mortality (HR: 1.23, *p* < 0.001) were both increased by pTURP.

## Discussion

Palliative transurethral resection of the prostate is not intended to treat prostate cancer or BOO, but to relieve lower urinary tract symptoms (LUTS) or bleeding caused by local progression of prostate cancer. Whether a patient suitable for pTURP or not is based on degree of secondary LUTS or bleeding, physical conditions and urodynamic parameters. We think it is very important a topic to discuss in the journal of Frontiers in Surgery. Previous studies showed that pTURP could benefit CSS and 5-year survival in mPCa and locally advanced PCa patients with BOO (2, 3). However, Krupski et al. hold the opposite views on pTURP and thought it as a feature of poor prognosis (4). The main reason was imbalance in disease severity between the two groups, particularly in terms of performance status and self-care ability (e.g., ECOG score), which would have led to a substantial selection bias in the prognostic analysis. ECOG score was considered to be a significant prognostic factor in castration-resistant prostate cancer (5).

The conclusion that pTURP reduced OS and CSS and increased mortality by the authors in patients with mPCa might be due to several shortcomings: (1) Patients in the Non-surgical group might not have BOO, or they might be unable to undergo surgical treatment due to poor general conditions, and only receive cystostomy or indwelling catheter. These two conditions would lead to different prognosis. (2) Two groups were balanced with respect to general conditions and oncology characteristics. However, data on prostate volume, degree of LUTS, and non-surgical treatment due to BOO were lacking. (3) Although the tumor stage and metastasis were balanced in PSM, the ECOG scores related to the prognosis of PCa patients were unclear.

Given the data of interest mentioned above were not included in the SEER database, and selection bias between groups might lead to inaccurate or even contrary conclusions. Therefore, recommendations from the database studies need to be re-examined for scientific validity and rationality, and further prospective studies will provide a higher level of evidence.

## Author contributions

CL and QY contributed to the writing of the manuscript. BS and XZ participated in the discussion of the application value of pTURP in mPCa. All authors contributed to the article and approved the submitted version.
